# Managed-Medicare Health Club Benefit and Reduced Health Care Costs Among Older Adults

**Published:** 2007-12-15

**Authors:** Huong Q Nguyen, Ronald T Ackermann, Matthew Maciejewski, Ethan Berke, Marsha Patrick, Barbara Williams, James P LoGerfo

**Affiliations:** Department of Biobehavioral Nursing and Health Systems, School of Nursing, University of Washington; Department of Medicine, Indiana University School of Medicine, Indianapolis, Indiana; Center for Health Services Research in Primary Care, Durham Veterans Administration Medical Center, and Division of Pharmaceutical Outcomes and Policy, University of North Carolina School of Pharmacy, Durham, North Carolina; Department of Community and Family Medicine, Dartmouth Medical School, Hanover, New Hampshire; U.S. Army, Combat Support Hospital, Yongsan, South Korea; Health Promotion Research Center, University of Washington, Seattle, Washington; Department of Medicine, School of Medicine, Department of Health Services, and Health Promotion Research Center, School of Public Health, University of Washington, Seattle, Washington

## Abstract

**Introduction:**

Our study was undertaken to determine the association between use of a health plan-sponsored health club benefit by older adults and total health care costs over 2 years.

**Methods:**

This retrospective cohort study used administrative and claims data from a Medicare Advantage plan. Participants (n = 4766) were enrolled in the plan for at least 1 year before participating in the plan-sponsored health club benefit (Silver Sneakers). Controls (n = 9035) were matched to participants by age and sex according to the index date of Silver Sneakers enrollment. Multivariate regression models were used to estimate health care use and costs and to make subgroup comparisons according to frequency of health club visits.

**Results:**

Compared with controls, Silver Sneakers participants were older and more likely to be male, used more preventive services, and had higher total health care costs at baseline. Adjusted total health care costs for Silver Sneakers participants and controls did not differ significantly in year 1. By year 2, compared with controls, Silver Sneakers participants had significantly fewer inpatient admissions (−2.3%, 95% confidence interval, −3.3% to −1.2%; *P* < .001) and lower total health care costs (−$500; 95% confidence interval, −$892 to −$106; *P* = .01]. Silver Sneakers participants who averaged at least two health club visits per week over 2 years incurred at least $1252 (95% confidence interval, −$1937 to −$567; *P* < .001) less in health care costs in year 2 than did those who visited on average less than once per week.

**Conclusion:**

Regular use of a health club benefit was associated with slower growth in total health care costs in the long term but not in the short term. These findings warrant additional prospective investigations to determine whether policies to offer health club benefits and promote physical activity among older adults can reduce increases in health care costs.

## Introduction

Despite the many benefits of physical activity, including better health, improved functioning, increased quality of life, and reduced mortality (1-4), approximately 25% of U.S. adults aged 65 or older engage in less than 10 minutes of moderate- or vigorous-intensity activities per week (5). Physical inactivity places an economic burden on the health care system and society as a whole (6-8). A longitudinal cohort study of people aged 54 to 69 showed that 2-year total health care expenditures were 7% lower for those who engaged in regular vigorous activity than for their sedentary counterparts (9). Another study estimated that health care costs for a previously sedentary adult aged older than 50 who engages in moderate physical activity at least 3 days per week can be reduced by $2200 over 2 years (10). As health care costs related to inactivity increase, more data are needed to assess the use of health policy and environmental change to promote health and reduce the impact of behavioral risks and chronic conditions (11-13).

Health plan promotion and support of physical activity via exercise programs as a coverage benefit has the potential to reach many people; 61% of Americans younger than 65 had employment-based health insurance in 2004 (14), and nearly 100% of Americans aged 65 or older had Medicare coverage. Two previous studies of a health plan-sponsored community-based group exercise program (EnhanceFitness) for Medicare Advantage (MA) plan enrollees found that adjusted 1-year health care costs were similar for participants and matched controls in a general population (15) and for a subgroup of members with diabetes (16).

The primary objective of this study was to determine whether the use of a health club benefit targeted to older adults was associated with a reduction in total health care costs. Our study extends the prior work in two ways. First, this study examines a different physical activity benefit (Silver Sneakers [SS]) in a larger population over a longer time frame, which enables us to determine whether participation is associated with change in health care use and costs over a 2-year period. Second, we used a larger sample to explore more fully the dose-response relationship between participation and total costs. Results from this study may provide evidence of the economic benefits of collaborations between health plans and health clubs to reduce physical inactivity by older adults.

## Methods

### Subject selection and eligibility

Our study was based at Group Health Cooperative of Puget Sound (GHC), a consumer-governed, mixed-model health maintenance organization with more than 500,000 members. We received administrative and claims data on 8473 members aged 65 or older who enrolled in the GHC MA plan, were continuously enrolled at GHC for at least 1 year before joining the program, and participated in SS from January 1, 1998, through December 31, 2003. Up to three GHC MA enrollees (n = 24,331) who never used the program were matched by age and sex to serve as controls for each SS participant. Participants and their matched controls were each assigned an index date representing the month that the participant first enrolled in SS. We excluded members who had less than 2 years of continuous enrollment after their index date, had missing cost data in any of the 3 years, had long-term care costs at baseline, or were unmatched SS participants or controls, which left 4766 SS participants and 9035 matched controls in our study for analysis.

The SS program provided the GHC MA enrollees access to selected local fitness centers in an unstructured format. Participants had access to conditioning classes designed for older adults, exercise equipment, a pool, a sauna, and other amenities that varied across facilities. A subcontractor administered the program and worked with the fitness centers. The GHC MA enrollees learned about the SS program from targeted mailings, a member benefits Web site, or their health care providers during routine preventive visits.

### Data sources

GHC administrative data, which have been used extensively in prior research (17,18), were the source of all utilization, cost, patient demographics, and other covariates. Cost variables were derived from the GHC cost accounting system, which integrates clinical information, units of service, and actual costs from the general ledger for 15 separate feeder systems. GHC identified all costs as either direct patient care costs or overhead costs. All overhead costs are fully allocated to individual patient care departments. Departments captured in the database included medical staff, nursing, pharmacy, laboratory, radiology, hospital inpatient, and community health services. Units of service were weighted by relative value units for ancillary departments, by technical relative value units for radiology, by College of Anatomical Pathology units for laboratory, and by visit length for outpatient visits. From this process, the precise cost for each unit of service delivered was then calculated, and costs were assigned to patients on the basis of units of service used. For example, primary care costs included all direct and indirect costs associated with visits or telephone calls by primary care or preventive medicine personnel that were related to direct patient care, preventive services, or risk factor reduction counseling.

The utilization outcomes we examined were for inpatient admissions, primary care visits, and specialty care visits (defined as obstetrics and gynecology services, cardiac diagnostics, diagnostic pathology, alternative medicine, and rehabilitative services). The cost outcomes we examined were for primary and specialty care costs, inpatient admission costs, and total health care costs. We selected primary care visits and costs because a more general outpatient cost summary was not available. Total health care costs were examined to provide an overall summary of the impact of SS participation on costs.

In the analysis, we controlled for covariates that might influence the economic outcomes that were available in GHC administrative data. These covariates included age, sex, baseline utilization or cost (as appropriate), inclusion on the GHC diabetes or heart registries, indication of arthritis on the outpatient visit problem list, patient risk, and a preventive services index. Comorbid conditions (arthritis, coronary artery disease, congestive heart failure, hypertension, depression, and diabetes) were ascertained from problem lists for outpatient visits according to *International Classification of Diseases, Ninth Revision, Clinical Modification* diagnostic codes (19). Patient risk was measured using RxRisk (20), a measure of chronic disease burden and comorbidity that was previously shown to have good predictive power for explaining odds of hospitalization (21) and total health care costs (15,16,22). RxRisk was calculated for each member on the basis of age, sex, and pharmacy utilization data for a 6-month period before the index date (20). Because members who use other preventive services may be more likely to participate in SS than are members who do not, we calculated a preventive services index to adjust for self-selection of health-oriented individuals into SS participation. The preventive services index was derived from the sum of the number of times a person received a fecal occult blood test, a flexible sigmoidoscopy, a screening mammogram, prostate cancer screening, an influenza vaccine, or a pneumococcal vaccine during the 2 years preceding the index date.

### Statistical analysis

We included all SS participants in the main analyses regardless of whether they made any visits to a health club over 2 years. We used two-tailed *t *tests and chi-square tests for unadjusted comparisons between SS participants and controls. We used multivariate ordinary least squares (OLS) regressions to determine differences in health care costs between SS participants and controls for 1 and 2 years after the index date while adjusting for age, sex, RxRisk, preventive services index, arthritis visits, inclusion in the health plan's heart or diabetes registries, and baseline use and costs. The results were similar to those obtained using generalized linear models with a gamma distribution and log-link function (23), so we present OLS results. Previous work suggests that using OLS regressions and large samples (≥500 observations) would yield unbiased estimates of absolute differences in use and cost data even when assumptions about normality and homoscedasticity are not met (24).

We performed exploratory dose-response analyses using OLS on the basis of the average number of health club visits during 2 years to determine incremental differences in total health care costs in members whose visits averaged fewer than 1 visit per week, 1 to fewer than 2 visits per week, 2 to fewer than 3 visits per week, and 3 or more visits per week. Average attendance was calculated by adding all health club visits during the 2 years and dividing by 104 weeks. SS participants who persisted with their visits to the health clubs for 2 years were compared with those who stopped using their physical activity benefit after the first year of SS enrollment. Because this study was interested primarily in differences in total health care costs between SS participants and controls and because subgroup analyses were purely exploratory, statistical tests were not adjusted for multiple comparisons.

To improve balance in observed covariates, we used propensity score (PS) adjustments in a sensitivity analysis (25,26) We estimated a logit model to generate each member's propensity of joining SS and entered PS as an additional covariate in our models. The inclusion of PS did not change the results of any of the models, so we present results from the simpler multivariate models.

All cost data were adjusted to 2003 dollars. Robust standard errors were used in all regressions. All statistical procedures were performed with Stata 9.0 (Stata Corporation, College Station, Texas). Institutional review boards at GHC and the University of Washington approved the study protocol.

## Results

### Unadjusted comparisons between SS participants and controls

Compared with controls, SS participants were slightly older, more likely to be male, had a lower chronic disease burden, used more preventive services, and had higher total health expenditures at baseline (Tables 1 and 2). A small percentage of members (2%) who signed up for the SS program never made a visit to a health club during the 2 years; another 2% did not visit a health club until the second year. The number of health club visits made by SS participants was 75 visits (median, 49; interquartile range, 11–120) in year 1 and 55 visits (median, 12; interquartile range, 0–89) in year 2.

The follow-up interval for all SS participants and controls was 2 years. In year 1, unadjusted total, inpatient admission, and specialty care costs were not different between SS participants and controls (Table 2). However, SS participants had more primary and specialty care visits (both, *P* < .05) and slightly fewer inpatient admissions than did controls (*P* = .02) in year 1. In year 2, SS participants had lower unadjusted total health care costs and fewer inpatient admissions than did controls (both, *P* < .01); unadjusted outpatient primary and specialty care utilization and costs were higher among SS participants (all, *P* < .01).

### Adjusted comparisons between SS participants and controls

In year 1, adjusted total health care costs were similar for SS participants and controls (+$2; 95% confidence interval [CI], −$341 to $344; *P* = .99) (Table 2). We observed a modest difference between SS participants and controls in inpatient admissions in the adjusted model (−1.0%; 95% CI, −2.1% to −0.1%; *P* = .05). SS participants made more primary and specialty care visits than did controls (both, *P* < .001).

By year 2, total health care costs were significantly lower for SS participants compared with controls (–$500; 95% CI, –$892 to –$106; *P* = .01). This difference in costs was mainly due to the fewer inpatient admissions among SS participants compared with controls (–2.3%, 95% CI, –3.3% to –1.2%; *P* < .001) and the slightly lower inpatient costs (–$270; 95% CI, –$533 to –$6; *P* = .05). SS participants made more primary and specialty care visits and incurred greater costs associated with primary care than did controls (all, *P* < .001).

### Exploratory adjusted dose-response analysis of health club use

SS participants were categorized according to the mean number of health club visits per week over 2 years: fewer than 1 visit per week, 1 to fewer than 2 visits per week, 2 to fewer than 3 visits per week, and 3 or more visits per week. We observed graded baseline differences in the proportion of women, RxRisk, mean preventive services index, and health care costs across the visit categories (results not shown). Adjusted models showed a significant threshold dose effect on total health care costs at year 2 (Figure). Compared with SS participants who averaged less than one visit per week, those who averaged 2 to fewer than 3 visits per week or 3 or more visits per week had similar reductions in total health care costs at year 2 (2 to <3 visits, –$1252; ≥3 visits, –$1309).

**Figure.**
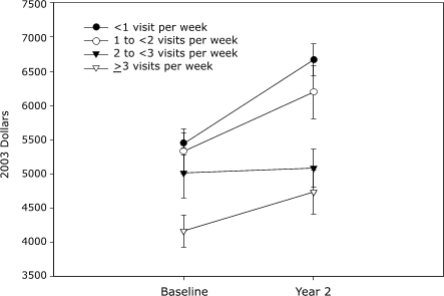
Adjusted total health care costs of Silver Sneakers (SS) participants in 2003 dollars, by mean number of health club visits per week for 2 years (top) and year 2 cost differences between categories of visits per week (bottom). SS participants (n = 4766) were categorized according to the mean number of health club visits per week over 2 years: fewer than 1 visit per week (n = 2778), 1 to fewer than 2 visits per week (n = 819), 2 to fewer than 3 visits per week (n = 593), and 3 or more visits per week (n = 576). Error bars indicate standard errors.

**Table:** 

Approximately 61% (n = 2902) of the SS participants continued to use their health club membership in the second year. The regular attendance of these *continuers *is reflected in their total number (SD) of health club visits. In year 1, continuers made 109 (84) visits, and in year 2, they made 89 (86) visits, higher than the average number of health club visits for the SS group as a whole. Although total health care costs at baseline were similar for both subgroups, members who did *not* continue health club attendance in year 2 (n = 1659), or *noncontinuers*, had significantly greater health service use in year 1. For example, more noncontinuers (11%) had an inpatient admission for all causes than did continuers (8%). In addition, noncontinuers had a mean (SD) of 5.7 (4.5) primary care visits and 3.5 (3.4) specialty care visits in year 1, compared with continuers, who had 5.1 (4.2) primary care visits and 3.2 (3.3) specialty care visits during the same year. Because we did not have access to data on whether disease burden increased in year 1, we compared the proportion of outpatient visits with new diagnostic codes between these two subgroups. In year 1, noncontinuers were more likely to receive new diagnostic codes on their problem list for arthritis (12.5%, noncontinuers vs 10.3%, continuers), cardiovascular disease (13.6%, noncontinuers vs 12.0%, continuers), diabetes (2.6%, noncontinuers vs 2.0%, continuers), and depression (8.1%, noncontinuers vs 5.6%, continuers).

## Discussion

We found that older Medicare beneficiaries who elected to use a health plan–sponsored physical activity benefit had significantly lower total adjusted health care costs (–$500) 2 years after the index start date compared with similar members who did not participate in the program. This cost difference is primarily a result of a lower number of SS members who had any inpatient admission combined with slightly lower inpatient care costs. We also found that greater use of the health club membership was associated with smaller increases in total health care costs from baseline to year 2. These results extend previous work suggesting that increased physical activity is associated with positive health outcomes, reduced mortality, and lower health care costs (2,9,10,27,28). Our estimates of cost reductions as a result of participation in the physical activity benefit were generally lower than those reported in other published studies that examined health care costs in relation to self-reported physical activity in older adults aged 59 to 69 (9) and older adults with a mean age of 63 (10). To our knowledge, this is the first study to examine the longitudinal effects of a health plan–sponsored physical activity benefit on health care costs and utilization for a large sample of older adults.

Notably, the cost of the health club benefit was included in the overall cost allocations in this study. Therefore, in constructing such benefits, payers will need to ensure that the benefit cost does not exceed savings and potential resources required to build incentives for regular participation. For older adults, greater access to fitness facilities may not necessarily encourage greater exercise participation. Recent figures from the health plan indicate that 25% of the eligible plan members were enrolled in the SS program in 2006. In any given month on average, however, only 28% of these enrollees actually visited the facilities at least once; that is, approximately 7% of the total plan membership actively used their benefit.

Many factors may influence a member's decision to make use of such benefits, such as awareness of the benefit, perceived accessibility to the fitness center or other exercise programs, and favorable attitudes and beliefs about exercise. Although a health care provider may mention the SS program to an older adult member during biennial preventive health visits, the health plan currently does not have formal follow-up processes in place to ensure that members are regularly encouraged to either continue with SS or other community-based exercise programs. Although efforts to increase physical activity in sedentary older adults can be resource-intensive and challenging, the financial returns for health plans that offer such physical activity benefits could be maximized with targeted efforts (29). Modest investments in improving the structure of SS to encourage consistent use of the physical activity benefit (e.g., 2 to 3 visits per week) could result in greater cost savings for the health plan.

Although a full economic analysis of the SS program that simultaneously accounts for costs and effects would be useful for health plans and decision makers (30), we did not have health status data for the GHC member population during this period. Insights can be gleaned from one recent cost-effectiveness analysis from the United Kingdom, which showed that a large-scale community-based program of exercise classes for older adults was effective in producing improvements in physical and mental health at an incremental cost of $33,637 per quality-adjusted life year gained (31). This cost estimate is remarkable, given that only 26% of the eligible study sample actually attended one or more class sessions — a factor that would have blunted estimates of health benefit, thereby making the cost-effectiveness ratio less favorable. More trials of such magnitude with rigorous cost-evaluations are clearly needed in the United States.

As is the case with all observational studies, we cannot completely exclude residual confounding or selection bias as an alternative explanation for our findings. SS participants engaged in more preventive screenings and had fewer illnesses than did controls. These differences may account for the lower health care costs regardless of participation in an exercise program. In addition, SS participants who were no longer using their health club benefit in year 2 had greater health service use in year 1 and indeed appeared to have developed new health problems that could have interfered with their continued participation. We did not have data on the types of exercise SS participants engaged in at the health clubs, nor did we have information on non-SS physical activity for all subjects. However, participation in other physical activity by controls would only have underestimated the differences in cost savings between the groups.

We attempted to control for both health status and health-seeking behavior by including a measure of chronic disease burden and a preventive services index in our regression models. We also included cost and utilization values before the index dates. By including these values as covariates in models with the same outcome at follow-up as the dependent variable, we addressed both potential confounding and differences between the groups at baseline. Participation in the SS program over time may have helped to increase older adults' functional capacity and self-efficacy to engage in other physical or social activities outside the program. This could partially explain why health care costs for participants did not differ from those for controls in year 1 but were significantly reduced in year 2, despite declines in the total number of SS visits over that time.

We showed that elective participation in a health club benefit, which had no impact on health care costs for older adults in the first year, was associated with lower total health care costs in the second year. Moreover, greater use of such benefits resulted in smaller increases in health care costs over 2 years. Given the limitations of the study design and methods, these findings need to be confirmed with randomized controlled trials to rule out the influence of self-selection and thereby provide more definitive evidence about the health and economic outcomes that result from health plans providing a health club benefit. These early results are encouraging, and if confirmed, may point to an effective strategy to increase physical activity among older adults.

## Acknowledgments

The authors acknowledge the support of Elizabeth Lin, MD, MPH, and staff at the Center for Health Studies of Group Health Cooperative of Puget Sound, Seattle, Washington.

This study was funded in part by the University of Washington Health Promotion Research Center (Centers for Disease Control and Prevention, U48-DP-000050) and grant number 1KL2RR025015-01 from the National Center for Research Resources (NCRR), a component of the National Institutes of Health (NIH) and the NIH Roadmap for Medical Research. The study's contents are solely the responsibility of the authors and do not necessarily represent the official views of the NCRR or NIH. Information on NCRR is available from http://www.ncrr.nih.gov/. Information on reengineering the Clinical Research Enterprise is available from http://nihroadmap.nih.gov/clinicalresearch/overview-translational.asp. Our funding sources were not involved in any aspect of the study design, conduct, or analysis, or with writing of the manuscript.

Dr Nguyen had full access to all the data in the study and takes responsibility for the integrity of the data and the accuracy of the data analysis. Other contributions include study concept and design: Nguyen, LoGerfo, Ackermann, Maciejewski; acquisition of data: Williams; analysis and interpretation of data: Nguyen, Ackermann, Maciejewski, Berke, Williams, LoGerfo; drafting of the manuscript: Nguyen, Ackermann, Maciejewski; critical revision of the manuscript for important intellectual content: Nguyen, Ackermann, Maciejewski, Berke, Patrick, Williams, LoGerfo; statistical analysis: Nguyen, Maciejewski, Williams.

## Figures and Tables

**Table 1:** Demographic and Health Characteristics of Participants and Controls, Silver Sneakers (SS) Program, Group Health Cooperative of Puget Sound, Seattle, Washington, 1998–2003

Characteristic	Controls (n = 9035)	SS Participants (n = 4766)	*P* Value^a^
**Demographics**			
Age, y, mean (SD)	72 (5)	73 (5)	.09
Women, n (%)	5987 (66)	3012 (63)	<.001
**Comorbidities,^b ^ n (%)**			
Arthritis	1450 (16.1)	990 (20.8)	<.001
Coronary artery disease	1087 (12.0)	593 (12.4)	.48
Inclusion in health plan's heart registry	1681 (18.6)	917 (19.2)	.36
Congestive heart failure	412 (4.6)	145 (3.0)	<.001
Hypertension	2233 (24.7)	1129 (23.7)	.18
Depression	816 (9.0)	458 (9.6)	.27
Diabetes	1427 (15.8)	620 (13.0)	<.001
Inclusion in health plan's diabetes registry	1413 (15.6)	618 (13.0)	<.001
**RxRisk,^c^ $, mean (SD)**	2557 (1676)	2416 (1443)	<.001
**Preventive services index,^d^ mean (SD)**	1.8 (1.7)	2.4 (1.8)	<.001

a Unadjusted comparisons were made using *t* test for unequal variance (continuous variables) or chi-square test (dichotomous variables).

b Comorbid conditions (arthritis, coronary artery disease, congestive heart failure, hypertension, depression, and diabetes) were ascertained from problem lists for outpatient visits according to *International Classification of Diseases, Ninth Revision, Clinical Modification *diagnostic codes (19).

c RxRisk is a measure of chronic disease burden and comorbidity (20) and is expressed as predicted 6-month costs in 2003 dollars. Higher costs represent higher comorbidity.

d Preventive services index is the sum of the number of times a person received preventive services in the 2 years preceding the index date. The following services were counted: fecal occult blood test, flexible sigmoidoscopy, screening mammogram, prostate cancer screening, influenza vaccine, and pneumococcal vaccine. Counts ranged from 0 to 8.

**Table 2:** Health Care Use and Health Care Costs 1 and 2 Years After Index Start Date, Silver Sneakers (SS) Program, Group Health Cooperative of Puget Sound, Seattle, Washington, 1998–2003

Use or Cost Measure per Year	Controls (n = 9035)	SS Participants (n = 4766)	Adjusted Mean Difference^a^ (95% Confidence Interval)	*P* Value
**Health Care Use**				
**No. (%) of people with an inpatient admission**				
Baseline	825 (9.1)	432 (9.1)	NA	NA
Year 1	984 (10.9)	454 (9.5)	–1.0% (–2.1% to –0.1%)	.05
Year 2	1129 (12.5)	471 (9.9)	–2.3% (–3.3% to –1.2%)	<.001
**No. of primary care visits per person**				
Baseline^b^	4.5 (5.0)	5.1 (4.3)	NA	NA
Year 1	4.7 (4.6-4.8)	5.3 (5.2-5.5)	0.40 (0.27-0.53)	<.001
Year 2	4.8 (4.7-4.9)	5.3 (5.2-5.4)	0.26 (0.13-0.40)	<.001
**No. of specialty care visits per person**				
Baseline^b^	2.7 (3.2)	3.2 (3.3)	NA	NA
Year 1	2.9 (2.8-3.0)	3.3 (3.2-3.4)	0.22 (0.11-0.33)	<.001
Year 2	3.0 (2.9 to 3.1)	3.4 (3.3-3.5)	0.25 (0.14-0.36)	<.001
**Health Care Costs per Person, $**				
**Total health care costs**				
Baseline^b^	4693 (7288)	5212 (8530)	NA	NA
Year 1	5687 (5486-5888)	5677 (5388-5966)	2 (–341 to 344)	.99
Year 2	6742 (6480-7003)	6155 (5843-6466)	–500 (–892 to –106)	.01
**Inpatient admission costs**				
Baseline^b^	1000 (4381)	1248 (6182)	NA	NA
Year 1	1391 (1268-1515)	1346 (1130-1561)	–32 (–279 to 214)	.80
Year 2	1803 (1644-1963)	1497 (1283-1711)	–270 (–533 to –6)	.05
**Primary care costs**				
Baseline^b^	788 (876)	911 (871)	NA	NA
Year 1	829 (810-849)	962 (937-988)	101 (70-133)	<.001
Year 2	875 (854-896)	983 (956-1010)	80 (46-113)	<.001
**Specialty care costs**				
Baseline^b^	716 (1254)	793 (1213)	NA	NA
Year 1	813 (783-843)	825 (792-857)	–14 (–58 to 29)	.51
Year 2	890 (860-922)	935 (895-975)	37 (–12 to 86)	.14

Values are expressed as either mean (SD) or mean (95% confidence interval). NA indicates not applicable.

a Adjusted mean difference is defined as the change from baseline in participants minus the change from baseline in controls. Differences were calculated using multivariate linear regression models with robust standard error estimates that controlled for age, sex, preventive services index, RxRisk (a measure of chronic disease burden and comorbidity [20]), indication of arthritis on the outpatient visit problem list, inclusion in the health plan's diabetes or heart registries, and baseline measures of health care utilization and cost.

b Two-tailed* t *tests and chi-square tests were used for unadjusted comparisons between controls and SS participants at baseline; *P* < .05.
